# Giant Proximal Right Colon Submucosal Hematoma Leading to a Large Bowel Obstruction

**DOI:** 10.7759/cureus.24599

**Published:** 2022-04-29

**Authors:** Arham Siddiqui, Hijab Ahmed, Muhammad H Nazim, Basem Soliman, Izi Obokhare

**Affiliations:** 1 General Surgery, Texas Tech University Health Sciences Center, Amarillo, USA

**Keywords:** abdominopelvic computed tomography, exploratory laparotomy, right hemicolectomy, large bowel obstruction, submucosal hematoma

## Abstract

A 41-year-old female with a previous history of chronic obstructive pulmonary disease (COPD) and polycythemia presented to the emergency department with worsening shortness of breath and cough which progressed to respiratory distress requiring mechanical ventilation. During her hospital stay, she developed abdominal distention followed by a fever and a four-point decrease in hemoglobin. A non-contrasted abdominopelvic CT scan was ordered which showed a very large retroperitoneal hematoma adjacent to the right colon with subtle active bleeding. Selective angioembolization of a distal segment of the right colic artery was performed by Interventional Radiology (IR) to achieve hemostasis and hemodynamic stability. Due to the persistent and worsening abdominal distention, a CT scan with contrast was ordered which clearly showed a submucosal hematoma in the region of the right colon extending from the hepatic flexure to the cecum. The hematoma was completely obstructing the proximal and mid ascending colon leading to a large bowel obstruction. Exploration of the abdomen showed severe bowel dilation, and frank ischemia of the hepatic flexure of the colon. Right hemicolectomy with primary ileocolonic anastomosis to evacuate the right retroperitoneal hematoma was subsequently performed. The patient was discharged on post-operative day 16 with no major complications.

## Introduction

Colonic hematomas occur at a lower frequency than small intestinal hematomas [1]. There are various causes of intestinal submucosal hematomas. For example, they may arise spontaneously due to anticoagulant therapy. A common complication of anticoagulant therapy is bleeding, with warfarin cited as the most common culprit [2]. Submucosal intestinal hematomas may also occur in response to other anticoagulant therapies such as low molecular weight heparin (LMWH). This complication is more commonly reported in children, but it can also be seen in elderly patients [3,4]. Additional causes of intestinal submucosal hematomas include blunt abdominal trauma, bleeding disorders, malignancies, and vasculitis [5]. Intestinal submucosal hematomas may lead to obstructive symptoms such as nausea, vomiting, and abdominal pain. Therefore, this must be part of the differential diagnosis for patients with anticoagulation presenting with abdominal pain. We present a case of a massive colonic submucosal hematoma on a patient on therapeutic anticoagulation.

## Case presentation

**Figure 1:**
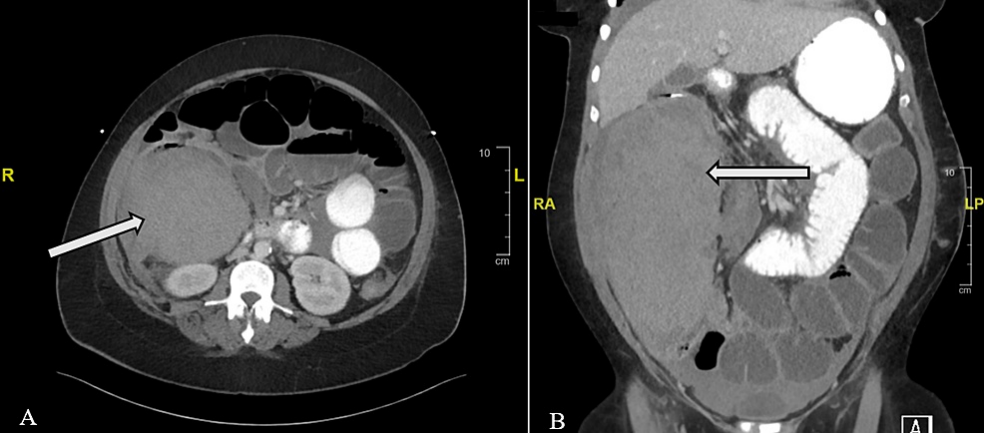
This figure shows coronal (A) and sagittal (B) CT scans with iopamidol contrast showing a massive colonic hematoma (see arrows). Subtle active bleeding is noted within the hematoma. It appears to be pushing into the dilated right hemicolon. There is also a moderate amount of free fluid in the abdomen and pelvis.

The patient is a 41-year-old African-American female with a previous history of chronic obstructive pulmonary disease (COPD), polycythemia, and hypertension. She presented to the emergency department with worsening shortness of breath and cough requiring intubation. After a few days of mechanical ventilation, she developed abdominal distention. This was managed nonoperatively with serial abdominal examinations, including an X-ray of the abdomen and pelvis. Imaging showed diffuse dilatation of the large bowel, suspected to be caused by adynamic ileus. However, the patient developed a persistent fever and a four-point decrease in hemoglobin with worsening abdominal pain. A non-contrast abdominopelvic computerized tomography (CT) scan revealed a very large retroperitoneal hematoma adjacent to the right colon with subtle active bleeding. Two units of packed red blood cells were ordered, and interventional radiology (IR) was urgently consulted. Selective angioembolization of a distal segment of the right colic artery was performed by IR to achieve hemostasis and hemodynamic stability. After successful embolization, a nasogastric tube was placed for decompression which actively drained fluid. In the following days, despite stable hemoglobin levels, the patient had worsening abdominal distention. A contrasted abdominopelvic CT scan was ordered which showed a very large submucosal hematoma in the region of the right hemicolon extending from the cecum to the hepatic flexure. The CT scan also showed free intra-abdominal blood (See Figure 1). The hematoma was completely obstructing the dilated right hemicolon leading to a large bowel obstruction. Physical examination was significant for right upper quadrant and right lower quadrant abdominal tenderness.

Exploration of the abdomen showed intraperitoneal blood, bowel dilation, and frank ischemia of the hepatic flexure. Right hemicolectomy with primary ileocolonic anastomosis and evacuation of the right retroperitoneal hematoma was performed. A surgical drain was left in the right upper quadrant. The patient was discharged on post-operative day 16 with no major complications.

## Discussion

A colonic submucosal hematoma is a rare finding; however, it is important to keep this in the differential as misdiagnosis can lead to untreated hemorrhage [6]. Understanding the risk factors for colonic submucosal hematoma can help establish the diagnosis. In this patient, an abdominopelvic CT scan with iopamidol contrast was ordered which led to the diagnosis. A CT scan without contrast may not be the most helpful and can result in inconclusive findings. A CT scan with oral contrast would assist in better visualization of the underlying colonic hematoma. The contrast used can be given orally or intravenously, however, in the setting of a complete bowel obstruction, it is recommended to administer contrast intravenously. This is because intravenous contrast can also help visualize gastrointestinal tract disorders such as Crohn’s disease or a colonic neoplasm. In the setting of a colonic submucosal hematoma, if a CT is not ordered, diagnosis may be limited to exploratory laparotomy or autopsy [5].

Management of colonic submucosal hematomas varies by the case presentation. A hematoma may resolve with non-operative management including discontinuing anticoagulant medications, administering total parenteral nutrition with intravenous hydration, and following up with CT [6]. Indications for an exploratory laparotomy can include severe compression or obstruction from the hematoma, hemodynamic instability suggestive of active hemorrhage, or intestinal perforation [7]. Moreover, there are various scenarios where it may be necessary to perform a colectomy. For instance, an urgent laparotomy with right hemicolectomy was performed in the setting of large bowel hematoma resulting in colonic perforation [8]. Another case reports a patient with both colonic adenocarcinoma and an intramural hematoma indicating right hemicolectomy to manage both diagnoses [6]. Finally, a hemicolectomy may be performed when the hematoma causes a bowel obstruction, as in the case of this patient. This was also reported in a patient with an idiopathic spontaneous large bowel hematoma obstructing the colonic bowel lumen requiring right hemicolectomy [9].

It is difficult to diagnose and treat colonic intramural hematomas, especially at the size seen in this patient. Patients with an intestinal hematoma will typically complain of abdominal pain and distention before symptoms of the hematoma obstructing the bowel appear [10]. Colonoscopy would show a bluish mucosa and erythematous formations in the submucosal layer in the involved colonic segment [11]. Situations, where there is potential perforation of the bowel, may dictate emergent laparotomy exploration. Abdominopelvic CT with iopamidol contrast will allow for definitive diagnosis and will typically show signs of wall thickening, diffuse fluid in the retroperitoneum, and evidence of a mass in the colon with or without obstruction [9]. Additionally, suspected ongoing bleeding in the presence of a colonic hematoma must be controlled to maintain hemodynamic stability in the patient and to limit the development of hemorrhagic shock. In our patient, this was achieved by angioembolization of the right colic artery.

## Conclusions

Early diagnosis and treatment of submucosal colonic hematomas is vital to a patient’s overall morbidity and mortality. This can be achieved by various means such as the expeditious order of an abdominopelvic CT with contrast when a colonic hematoma is suspected. The patient in this case report had a medical history of COPD, polycythemia, obesity, hypertension, acute hypoxemic respiratory failure, and was on anticoagulation. Imaging can facilitate recognition of the disease process and allow for prompt management and treatment.
